# Atomic Layer Deposition of GdCoO_3_ and Gd_0.9_Ca_0.1_CoO_3_

**DOI:** 10.3390/ma13010024

**Published:** 2019-12-19

**Authors:** Marion Duparc, Henrik Hovde Sønsteby, Ola Nilsen, Anja Olafsen Sjåstad, Helmer Fjellvåg

**Affiliations:** Centre for Materials Science and Nanotechnology, Department of Chemistry, University of Oslo, 0315 Oslo, Norway; m.j.l.duparc@smn.uio.no (M.D.); h.h.sonsteby@kjemi.uio.no (H.H.S.); ola.nilsen@kjemi.uio.no (O.N.); a.o.sjastad@kjemi.uio.no (A.O.S.)

**Keywords:** gadolinium cobaltites, atomic layer deposition, β-diketonates, ozone, preferential crystal growth orientation, high-aspect-ratio substrate

## Abstract

Thin films of the catalytically interesting ternary and quaternary perovskites GdCoO_3_ and Gd_0.9_Ca_0.1_CoO_3_ are fabricated by atomic layer deposition using metal β-diketonates and ozone as precursors. The resulting thin films are amorphous as deposited and become single-oriented crystalline on LaAlO_3_(100) and YAlO_3_(100/010) after post-annealing at 650 °C in air. The crystal orientations of the films are tunable by choice and the orientation of the substrate, mitigated through the interface via solid face epitaxy upon annealing. The films exhibit no sign of Co^2+^. Additionally, high-aspect-ratio Si(100) substrates were used to document the suitability of the developed process for the preparation of coatings on more complex, high-surface-area structures. We believe that coatings of GdCoO_3_ and Gd_1−*x*_Ca*_x_*CoO_3_ may find applications within oxidation catalysis.

## 1. Introduction

Rare-earth element perovskites with the formula ABO_3_ (A = alkaline/ rare-earth element, B = 3d–5d transition metal) have received much attention in the field of heterogeneous catalysis [1,2,3]. The catalytic activity of these materials relates to the nature of the B-site element [2]. In addition, the partial substitution of the A-site alkaline/rare-earth element with a lower valency cation (typically Ca or Sr) may result in oxygen nonstoichiometry, which in turn induces specific effects on the catalytic performance [3]. Encouraging results for catalytic oxidation reactions have been obtained with LaCoO_3_ and La_1−x_A’_x_CoO_3_ (A’ = Ca or Sr) [1,3]. However, the basicity of lanthanum makes such catalysts vulnerable to detrimental volume expansion due to lanthanum oxide hydration upon reaction with air and moisture [4,5]. Preliminary bench-scale catalyst performance tests of bulk Gd_1−*x*_Ca*_x_*CoO_3_ for ammonia oxidation show comparable catalytic performance to the corresponding La-based system, but without the undesired degradation of the catalysts due to hydroxide formation upon temperature cycling in the processing atmosphere [6]. We currently focus on GdCoO_3_-based catalysts of relevance for the ammonia slip reaction (i.e., the oxidation of minute quantities of NH_3_ in a process stream into nitrogen and steam), owing to the lower basicity, and thus improved resistance towards hydration, of such Gd-containing compounds in realistic processing environments [7,8]. Notably, we also explore deposition routes for Ca-substituted variants, providing means for oxygen vacancies.

Recent literature underlines the pertinence of using atomic layer deposition (ALD) in the design and study of coatings for heterogeneous catalysis [9,10,11]. The sequential nature of the ALD technique inherently rules out any gas phase reactions, and the self-limiting nature of the processes leads to controllable and reproducible synthesis of morphologically and chemically uniform materials [12,13,14]. A major advantage of ALD compared to conventional thin-film synthesis routes like sputtering or CVD is the possibility of obtaining high-surface-area supported catalysts by depositing chemically uniform thin films of the active phase on a high-surface-area support [15,16]. This is enabled by the self-limiting mechanism that allows for deposition beyond the line-of-sight.

ALD processes have been developed and reported for a wide variety of oxides, including around 30 functional perovskites [17,18]. The development of ALD processes for ternary and quaternary oxides has recently gained attention due to their high potential in a range of applications, such as ferroelectrics, photovoltaics, and battery technology [18,19]. However, owing to the complexity of multi-cation deposition, the available ALD processes for quaternary oxides are still limited to a few systems [20]. To the best of our knowledge, no reports have been published on the preparation of ALD films of GdCoO_3_ or the substituted variants thereof.

ALD of LaCoO_3_ using β-diketonates and ozone was reported in 1997 by Seim et al. [21]. No reports were made of any structural or functional characterization of the product films. More recently, ALD of the quaternary La_1−*x*_Sr*_x_*CoO_3-δ_ system for the composition range 0.3 < *x* < 0.7 was achieved by Ahvenniemi et al., using the same type of process [20]. One of the challenges of introducing cobalt in complex oxide ALD is catalytic decomposition of ozone and the metal‒organic precursors by CoO*_x_* species. This challenge can be overcome by tuning the precursor flux and precursor sequence, similar to recent reports on the deposition of lanthanum cuprate, for which CuO_x_ species exhibit the same detrimental catalytic precursor decomposition [22].

In this work we report for the first time the controlled thin-film growth and characterization of the ternary GdCoO_3_ and quaternary Gd_1−*x*_Ca*_x_*CoO_3_ rare-earth cobaltites using β-diketonates and ozone as precursors on flat and high-aspect ratio substrates. The current investigation is a step towards the growth and tailoring of highly selective complex thin films for heterogeneous catalysis.

## 2. Experimental

### 2.1. ALD and Precursors

All thin films were deposited in a F-120 Sat reactor (ASM Microchemistry, Helsinki, Finland) at a reactor temperature of 300 °C, unless otherwise stated. The temperature was chosen to comply with applicable temperatures for the binary oxide processes. Nitrogen was used as a purging gas, supplied from gas cylinders (99.999%, Praxair Norway, Oslo, Norway) and run through a Mykrolis purifier (Avantor Fluid Handling LLC, Devens, MA, USA) to remove oxygen and water impurities. The purging gas was maintained at a 300 cm^3^ min^−1^ flow rate, giving an operating pressure of 2.6 mbar throughout the process.

Co(thd)_2_ (99.9+%, Volatec, Porvoo, Finland), Gd(thd)_3_ (99.9%, Strem Chemicals Inc., Kehl, Germany) and Ca(thd)_2_ (99.9+%, Volatec) were used as cation sources, maintained in open boats in the reactor at 115 °C, 140 °C, and 198 °C, respectively (thd = 2,2,6,6-tetramethyl-3,5-heptanedionate). All precursors were re-sublimated before use to enhance purity. O_3_ was used as the oxygen source, made from O_2_ gas (99.6%, Praxair Norway) with an In USA (AC-2505) ozone generator producing 15 mass% O_3_ in O_2_. Pulse durations were set to 1.5 s for all metal precursors, whereas ozone was pulsed for 5 s subsequent to Co(thd)_2_ and 3 s subsequent to Gd(thd)_3_ and Ca(thd)_2_ pulses. All purge durations were set to 2 s. The pulse and purge durations were chosen in agreement with previous reports of self-limiting growth using these precursors in similar reactor infrastructures.

### 2.2. Substrates and Annealing

Films were routinely deposited on 1 × 1 cm^2^ Si(100) for characterization of thickness, whereas 3 × 3 cm^2^ Si(100) substrates were used for compositional analysis with X-ray fluorescence (XRF) and for investigating any thickness gradients. Selected films were deposited on 1 × 1 cm^2^ LaAlO_3_ (100)_pseudocubic_ (LAO, MTI Corp., Richmond, CA, USA), YAlO_3_ (100) (MTI Corp.) and YAlO_3_ (010) (YAP, MTI Corp.) for the facilitation of epitaxial growth. The investigation of conformality on high-aspect-ratio substrates was carried out on silicon substrates with parallel grooves of 20 μm depth and 10 μm width (SINTEF IKT made by reactive ion etching with the Bosch process).

The selected films were annealed at 650 °C for 30 min in 1 atm air in an OTF-1200X rapid thermal processing (RTP) furnace (MTI Corp., Richmond, CA, USA) to facilitate crystallization prior to structural investigation.

### 2.3. Characterization

Film thickness was routinely studied using a J. A. Woollam alpha-SE spectroscopic ellipsometer (J.A. Woollam Co., Lincoln, NE, USA) in the wavelength range 390–900 nm. A Cauchy function was successfully used to model the collected data.

X-ray diffraction (XRD) measurements were used to investigate the out-of-plane crystalline orientation of the thin films on single crystal substrates. Symmetric θ-2θ-scans were carried out on a Bruker AXS D8 Discover diffractometer (Bruker AXS, Karlsruhe, Germany) equipped with a LynxEye strip detector (Bruker AXS) and a Ge (111) focusing monochromator, providing CuKα_1_ radiation.

Chemical composition was analyzed using a Panalytical Axios Max Minerals XRF system (Malvern Panalytical, Malvern, UK) equipped with a 4 kW Rh tube. Omnian and Stratos options were employed for standardless measurements of thin film cation composition.

The chemical state of the cations, particularly cobalt, was investigated by X-ray photoelectron spectrometry (XPS) using a Thermo Scientific Theta Probe Angle-Resolved XPS system (ThermoFisher Scientific, Waltham, MA, USA). The instrument was run with a standard Al Kα source (hν = 1486.6 eV), and the analysis chamber pressure was maintained on the order of 10^−8^ mbar. Pass energy values of 200 eV and 50 eV were employed for survey scans and detailed scans, respectively. The data were corrected for any drift by setting the binding energy for adventitious carbon to 284.8 eV. Data treatment and fitting were performed within the Avantage software suite (ThermoFisher Scientific). The background was fitted to a Shirley-type pseudostep function.

Cross section SEM images of the deposited films were obtained using a Hitachi SU8230 SEM (Hitachi, Krefeld, Germany) with a cold cathode field emission electron gun. The total voltage was set to 2 kV and the films were imaged by means of secondary and back-scattered electrons. 

## 3. Results and Discussion

### 3.1. Deposition of GdCoO_3_

The development of ternary deposition processes typically requires insight into the individual growth behavior of the binary components. ALD of Co_3_O_4_, using Co(thd)_2_ as a precursor and ozone as the oxidizing agent, was established by Klepper et al. in the 114–307 °C temperature range, with a growth per cycle (GPC) of ≈ 0.20 Å/cycle at 300 °C [23]. For a similar Gd(thd)_3_-based deposition process, Niinistö et al. reported self-limiting growth for Gd_2_O_3_ films in the range from 250 to 300 °C, with a GPC of ≈ 0.30 Å/cycle at 300 °C [24]. Our attempts at deposition of the same binary processes gave reproducible GPCs of 0.16 Å/cycle and 0.37 Å/cycle for the formation of CoO_x_ and Gd_2_O_3_, respectively, with no observed thickness gradients. 

Based on these results, a series of (Gd, Co)-oxide films were deposited at 300 °C. The Gd(thd)_3_: Co(thd)_2_ pulsed ratio was varied systematically to identify the conditions required to obtain the desired deposited stoichiometry of GdCoO_3_. We employed a super cycle approach with a general super cycle, given as:

(1) $$n\;\times \{m\;\times [Gd\left(thd{)}_{3}+O_{3}\right]+l\;\times [Co\left(thd{)}_{2}+O_{3}\right]\},$$

where *n*, *m*, and *l* were varied to achieve the desired cation ratio.

**Figure 1:**
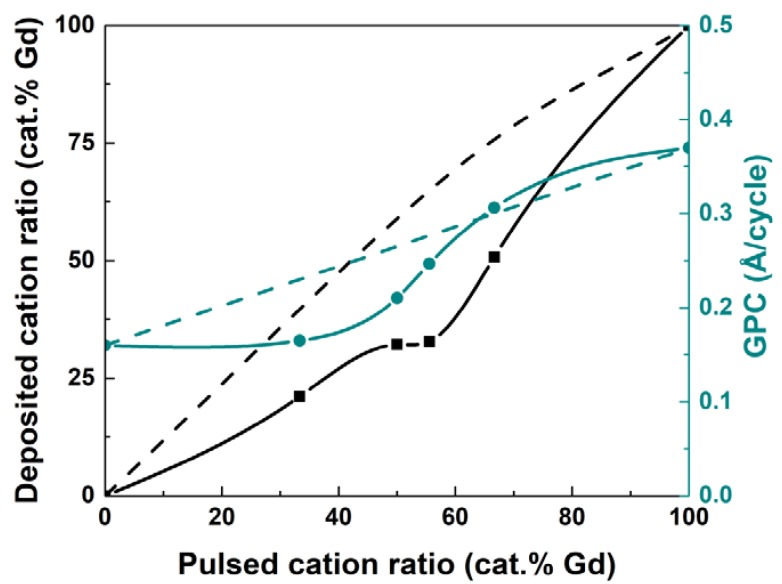
Deposited cation ratio (cat.% Gd, as measured by XRF) and GPC as a function of the pulsed cation ratio (cat.% Gd) for (Gd, Co)-oxide films deposited at 300 °C. The dotted lines refer to deposited cation ratio (cat.% Gd) and GPC as a function of the pulsed cation ratio (cat.% Gd) for Gd_2_O_3_ film.

Figure 1 shows the deposited cation ratio for Gd (cat.% Gd) of the obtained film and the GPC as a function of the pulsed cation ratio (cat.% Gd) at 300 °C. The relative amount of deposited Gd increases from 2 to 51 cat.% Gd in the explored pulsed cation range of 33–67 cat.% Gd. The concentration of Gd in the deposited film consistently increases with increasing amounts of pulsed Gd(thd)_3_, except for the plateau interval observed between 50 and 56 cat.% Gd pulsing ratio, where the Gd concentration in the product takes a constant value at around 32 cat.%. We note that the desired Gd:Co ratio of close to unity is obtained for 67 cat.% of pulsed Gd. The GPC of the deposited films at 300 °C increases smoothly with an increased fraction of Gd pulses, in accordance with the higher GPC of Gd_2_O_3_, see Figure 1. However, an excess of Gd pulses must be applied to achieve stoichiometric GdCoO_3_. We do observe a small reduction in overall GPC (0.24 Å/cycle) as compared to a linear combination of the binary oxides [(0.37 + 0.16)/2 = 0.27 Å/cycle], possibly due to either inhibition of growth from Gd(thd)_3_ on Co-O* surfaces or by increased growth from Co(thd)_2_ on Gd-O* surfaces, or most likely a combination of both judging from the dependency of pulsed to deposited composition in Figure 1. This is an effect seen in several ALD processes, such as reported earlier by our group in the case of LaAlO_3_ [25]. The GPCs obtained at 300 °C for (Gd, Co)-oxides are in good agreement with the results of Seim et al., who reported an average GPC of 0.35 Å/cycle for LaCoO_3_ at 350 °C following a similar β-diketonate and ozone deposition process [21].

### 3.2. Deposition of Gd_1−x_Ca_x_CoO_3_

**Figure 2:**
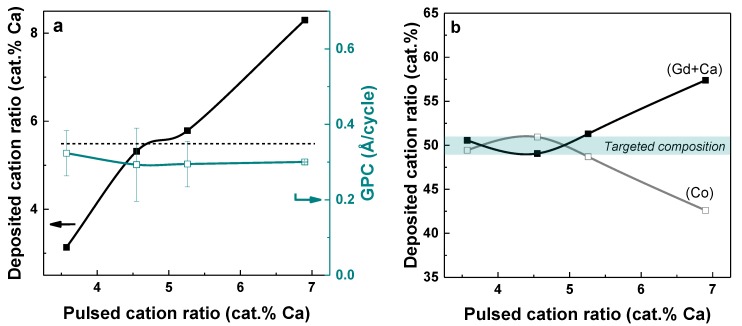
(**a**) Deposited cation ratio (cat.% Ca, as measured by XRF) and (**b**) GPC deposited cation ratio (cat. % (Gd + Ca) and Co, as measured by XRF) as a function of the pulsed cation ratio (cat.% Ca) for (Gd, Ca, Co) films deposited at 300 °C. The dotted line indicates the targeted Ca deposited concentration.

Based on the results obtained for the ternary (Gd, Co)-oxide system, the quaternary (Gd, Ca, Co)-oxide system was explored in an attempt to target products with the Gd_0.9_Ca_0.1_CoO_3_ composition. ALD was carried out at 300 °C following an identical process as for ternary (Gd, Co)-oxide films, with the essential modification of substituting a number of Gd(thd)_3_-pulses with Ca(thd)_2_-pulses. The [Gd(thd)_3_ + Ca(thd)_2_]: Co(thd)_2_ pulsed ratio was maintained at 2:1 in order to keep the deposited (Gd + Ca): Co atomic ratio close to unity. The Ca pulsed ratio, using Ca(thd)_2_ as precursor, was varied from 3 to 7 cat.%. Figure 2a shows the deposited cation ratios and the GPC as a function of the Ca pulsed ratio (cat.%) for depositions at 300 °C. The Ca content in the films correlates fairly well with the relative amount of Ca pulses. In a few experiments deviating behavior was observed, which reflects the challenge of controlling the simultaneous growth of three different cation species [26]. However, quite a stable growth situation was obtained for the range around 4–5 cat.% Ca. The A-site (Gd + Ca): B-site (Co) stoichiometry was analyzed as function of the relative amount of Ca-pulses (Figure 2b). With the current pulsing strategy, the target (Gd + Ca): Co ratio close to unity is obtained for films deposited at a Ca pulsed ratio between 3.5 and 5 cat.%. The targeted composition Gd_0.9_Ca_0.1_CoO_3_ is obtained for a Ca pulsed ratio of 4.5 cat.%, for which an equiatomic ratio is maintained between the perovskite A- and B-sites. 

### 3.3. Characterization of GdCoO_3_ and Gd_0.9_Ca_0.1_CoO_3_ Thin Films

#### 3.3.1. X-Ray Diffraction (XRD)

**Figure 3:**
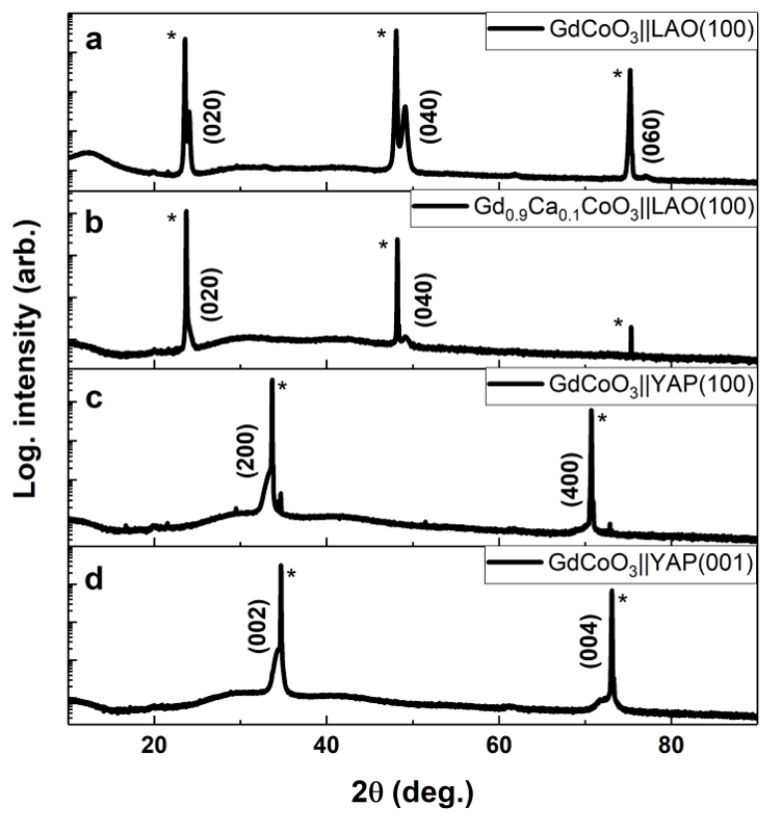
XRD patterns of (**a**) 30 nm GdCoO_3_ and (**b**) 30 nm Gd_0.9_Ca_0.1_CoO_3_ films grown on LAO(100), (**c**) 30 nm GdCoO_3_ grown on YAP(100) and (**d**) 30 nm GdCoO_3_ grown on YAP(001), post-annealed for 30 min at 650 °C. Bragg reflections originating from the substrate are marked with a star; film reflections are marked with their designated plane of reflection.

The as-prepared GdCoO_3_ and Gd_0.9_Ca_0.1_CoO_3_ films deposited at 300 °C are X-ray amorphous. Crystallization is achieved upon annealing at 650 °C for 30 min in air on LAO and YAP single crystals, resulting in preferential orientation depending on the substrate type and orientation. Figure 3a,b shows XRD patterns of post-annealed GdCoO_3_ and Gd_0.9_Ca_0.1_CoO_3_ films deposited on LAO(100)_pc_. The diffractograms for the crystalline films on LAO(100)_pc_ can be indexed as orthorhombic GdCoO_3_ (*Pbnm*, SG# 62; Z = 4) with a preferred (010) growth orientation. The orthorhombically distorted GdCoO_3_ perovskite relates to the ideal cubic perovskite structure (*Pm-3m*; Z = 1) as *a_o_* = $\sqrt{2}$ × a_c_, *b_o_* = 2 × *b_c_*, *c_o_* = $\sqrt{2}$ × c_c_ with dimensions *a_o_* = 5.380 Å, *b_o_* = 7.437 Å and *c_o_* = 5.210 Å. The (rhombohedral) LAO substrate exhibits a pseudo cubic structure *a_c_* = 3.79 Å (note $\sqrt{2}$ × *a_c_* = 5.36 Å). The growth of GdCoO_3_-based perovskites onto LAO is favored in the (010) orientation as the *a*- and *c*-axis of the film match the diagonals of the cube faces of the substrate. In this configuration, GdCoO_3_ will experience a lattice expansion of 2.5% in order to match the diagonal by diagonal area of the LAO substrate (A_LAO_ = *a_c_^2^* = 28.73 Å^2^ and A_GCO_ = *a_o_* × *c_o_* = 28.02 Å^2^). The position of the (020) and (040) reflections indicate that *b_GCO||LAO(100)_* = 7.42 Å (strain−0.2%), which indicates a small compression compared to the theoretical orthorhombic structure. This is in good agreement with the expected expansion in *a*. We used Scherrer’s formula on the well-defined GdCoO_3_ (040) reflection (Supporting Figure S1) to estimate a crystallite size of 24.8 nm, which indicates that the crystallites traverse from the substrate to the film surface. A higher degree of crystallinity is observed for GdCoO_3_, which exhibits sharper and more intense (020) and (040) reflections than Gd_0.9_Ca_0.1_CoO_3_. This is in good agreement with Bretos et al., who reported a slower crystallization process for Ca-substituted perovskites [27].

Figure 3c,d shows the measured XRD patterns from crystalline GdCoO_3_ films deposited on YAP (100) and YAP (001), respectively, after post annealing at 650 °C in air for 30 min. Both YAP and GdCoO_3_ are orthorhombic perovskites and exhibit quite similar unit cell dimensions; for YAP, *a_YAP_* = 5.330 Å, *b_YAP_* = 7.375 Å and *c_YAP_* = 5.180 Å. GdCoO_3_ deposited on YAP(001) grows with a preferred (001) orientation, whereas GdCoO_3_ deposited on YAP(100) exhibits a preferential (100) growth orientation. Thus, the preferential (001) growth orientation of GdCoO_3_ onto an oriented YAP(001) substrate is favored due to a minimized lattice compressive stress of 1.7% in this configuration (V_YAP(001)_ = *a_YAP_* × *b_YAP_* = 39.31 Å^2^ and V_GCO_ = *a_o_* × *b_o_* = 39.97 Å^2^). On the other hand, a (100) orientation of the substrate results in the growth of GdCoO_3_ in a preferential (100) orientation with a lattice compression of 1.2% (V_YAP(100)_ = *b_YAP_* × *c_YAP_* = 38.20 Å^2^ and V_GCO_ = *b_o_* × *c_o_* = 38.64 Å^2^). The close lattice match means that the film reflections are observed as a broadening of the substrate peaks, making it difficult to analyze the diffraction in terms of crystallite size or strain. The Gd_0.9_Ca_0.1_CoO_3_ thin films deposited on YAP substrates exhibited too poor a crystallinity, even after annealing, to be properly indexed (see Supplementary Figure S2).

By use of this appropriate selection of substrates, we have demonstrated the preferred crystallization along all three crystallographic axes. The effect of surface structure on catalytic activity is well known, so the ability to select the growth orientation of crystalline films may be of high importance.

The physical properties of gadolinium cobaltites depend, inter alia, on the temperature and cation substitutions, type, and concentration, which in turn may have a profound effect on the catalytic performance. For instance, an expanded lattice triggered by the substrate may stabilize the high-spin Co(III) configuration at temperatures lower than 800 K, i.e., the transition temperature for bulk GdCoO_3_ [28]. Such scenarios are interesting from an ALD perspective, since key physical and chemical performance properties may be tuned by the choice of appropriate lattice-matching substrates.

#### 3.3.2. XPS

**Figure 4:**
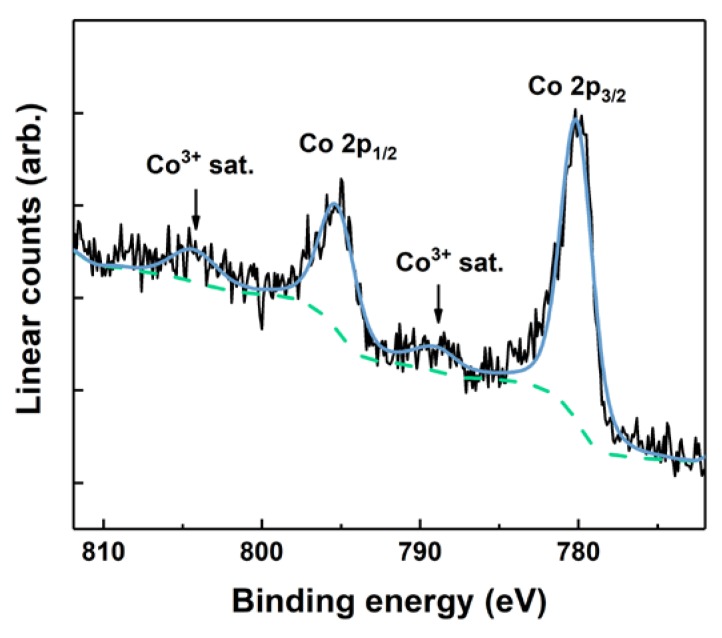
Co 2p XPS of 30 nm GdCoO_3_ thin films on LaAlO_3_ (100), with no observation of Co^2+^. The black line is the recorded data, the green line is the background, and the light blue line is the total fit.

Detailed XPS spectra close to the Co 2p binding energies were collected to identify the chemical state of cobalt in the films (Figure 4). Previous reports of ALD-grown cobalt oxide using β-diketonates and ozone indicated a mixed 2^+^/3^+^ valence. The presence of Co^2+^ could indicate detrimental inclusions of Co_3_O_4_ in the grown films. Co^2+^ can be identified by an intense shake-up satellite feature at around 786 eV, whereas the Co^3+^ satellite is shifted towards 790 eV. Currently, we only observed Co^3+^ satellite features, indicating that the films are dominated by GdCoO_3._ Based on the data and the complexity of Co XPS, however, we cannot rule out that some Co^2+^ is present in the films. Survey spectra and detailed scans of O 1 s and C 1 s can be found in the Supplementary Materials (Figure S3).

#### 3.3.3. Deposition on a High-Aspect-Ratio Substrate

**Figure 5:**
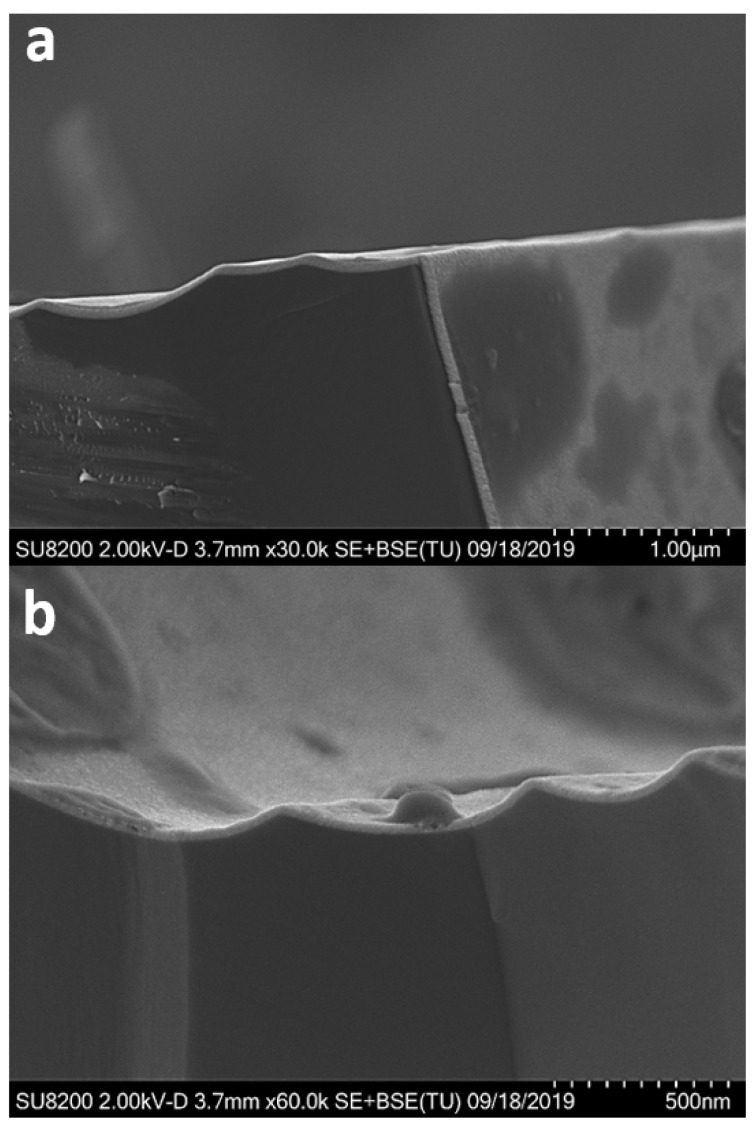
SEM cross-sectional images of GdCoO_3_ deposited on a trench Si wafer: (**a**) top trench view; (**b**) bottom trench view.

The ability to deposit catalytically active complex oxides on high aspect ratios is of high importance. This can, e.g., enable the coating of mesoporous γ-alumina, and thereby provide catalysts with a significantly enhanced surface area compared to nanoparticles (10–100 nm) obtained from wet chemical synthesis and/or ball milling. The conformality of the two gadolinium cobaltite-based films was investigated by applying the presented deposition process onto high-aspect-ratio substrates. Figure 5 shows cross section images of a GdCoO_3_ film deposited on a high-aspect-ratio trench Si wafer. The film is conformally deposited on all surfaces of the substrate. As shown in Figure 5b, the bottom of the trench is characterized by the presence of agglomerates, possibly resulting from turbulence during growth and/or the preparation of cross section SEM samples. 

## 4. Conclusions

We have developed an ALD process for crystalline and homogeneous Gd‒Ca‒Co‒O thin films on three different substrates, which provides a route towards coatings with potential application within catalysis. The good crystallinity of the obtained films gives insight into the crystal structure of the product and orientation of crystallization, which is essential since catalytic performance depends on key parameters connected with the structure, chemistry, and electronic states of exposed surfaces. These features are shown to be tuned by appropriate choices of ALD precursors, substrates, and deposition/annealing conditions. The proof of concept of depositing conformal Gd‒Ca‒Co‒O films on high-aspect-ratio substrates is important, since practical applications in catalysis would require high surface areas.

The gadolinium cobaltite-based catalysts represent a particularly interesting system, not only with respect to catalysis, but also to physical properties. For instance, an expanded lattice triggered by the substrate may possibly stabilize a high-spin Co(III) state. Such scenarios suggest that ALD can be used to tune resulting properties by means of appropriate lattice-matching substrates. 

## Supplementary Materials

The following are available online at https://www.mdpi.com/1996-1944/13/1/24/s1: Figure S1: XRD patterns of 30 nm Gd_0.9_Ca_0.1_CoO_3_ films grown on (a) YAP(100) and (b) YAP(001), post-annealed for 30 minutes at 650 °C. Figure S2: XRD pattern of the GdCoO_3_ (040) reflection of as deposited (black) and annealed (green) 30 nm films grown on LAO (100)_pc_, used for Scherrer analysis of crystallite size. Figure S3: (a) XPS of C 1s, (b) XPS of O 1s and (c) Survey spectra showing identification of Gd, Co, O and carbon species.
