# Supplementary material for: Atomic Layer Deposition of GdCoO3 and Gd0.9Ca0.1CoO3
Source: Materials (Basel). 2019 Dec 19;13(1):24. doi: 10.3390/ma13010024 (PMC6982051; doi:10.3390/ma13010024)
Supplement: Supplementary file 1 [file materials-13-00024-s001.pdf]

*materials*

*Supplementary Materials*

# Atomic Layer Deposition of $GdCoO_3$ and $Gd_{0.9}Ca_{0.1}CoO_3$

**Marion Duparc, Henrik Hovde Sønsteby, Ola Nilsen, Anja Olafsen Sjåstad and Helmer Fjellvåg**

Centre for Materials Science and Nanotechnology, Department of Chemistry, University of Oslo, 0315 Oslo, Norway; m.j.l.duparc@smn.uio.no (M.D.); h.h.sonsteby@kjemi.uio.no (H.H.S.); ola.nilsen@kjemi.uio.no (O.N.); a.o.sjastad@kjemi.uio.no (A.O.S.); helmer.fjellvag@kjemi.uio.no (H.F.)

* Correspondence: helmer.fjellvag@kjemi.uio.no

Received: 16 November 2019; Accepted: 16 December 2019; Published: date

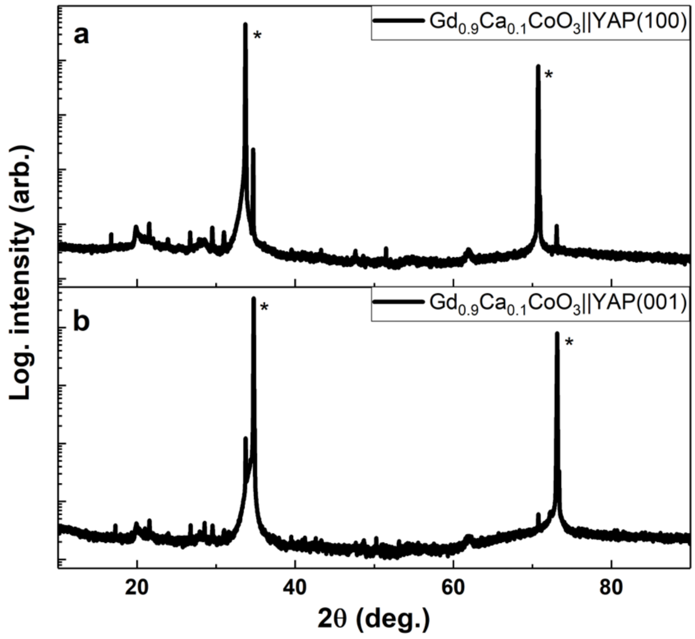


**Figure S1.** XRD patterns of 30 nm $Gd_{0.9}Ca_{0.1}CoO_3$ films grown on (**a**) YAP(100) and (**b**) YAP(001), post-annealed for 30 minutes at 650 °C. Identified Bragg-reflections originating from the substrate are marked with a star.

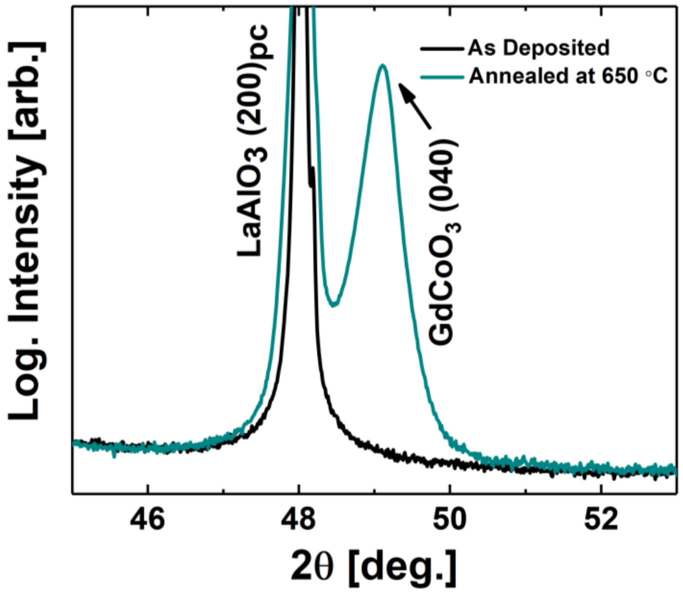

**Figure S2.** XRD pattern of the $GdCoO_3$ (040) reflection of as deposited (**black**) and annealed (**green**) 30 nm films grown on LAO $(100)_{pc}$, used for Scherrer analysis of crystallite size.

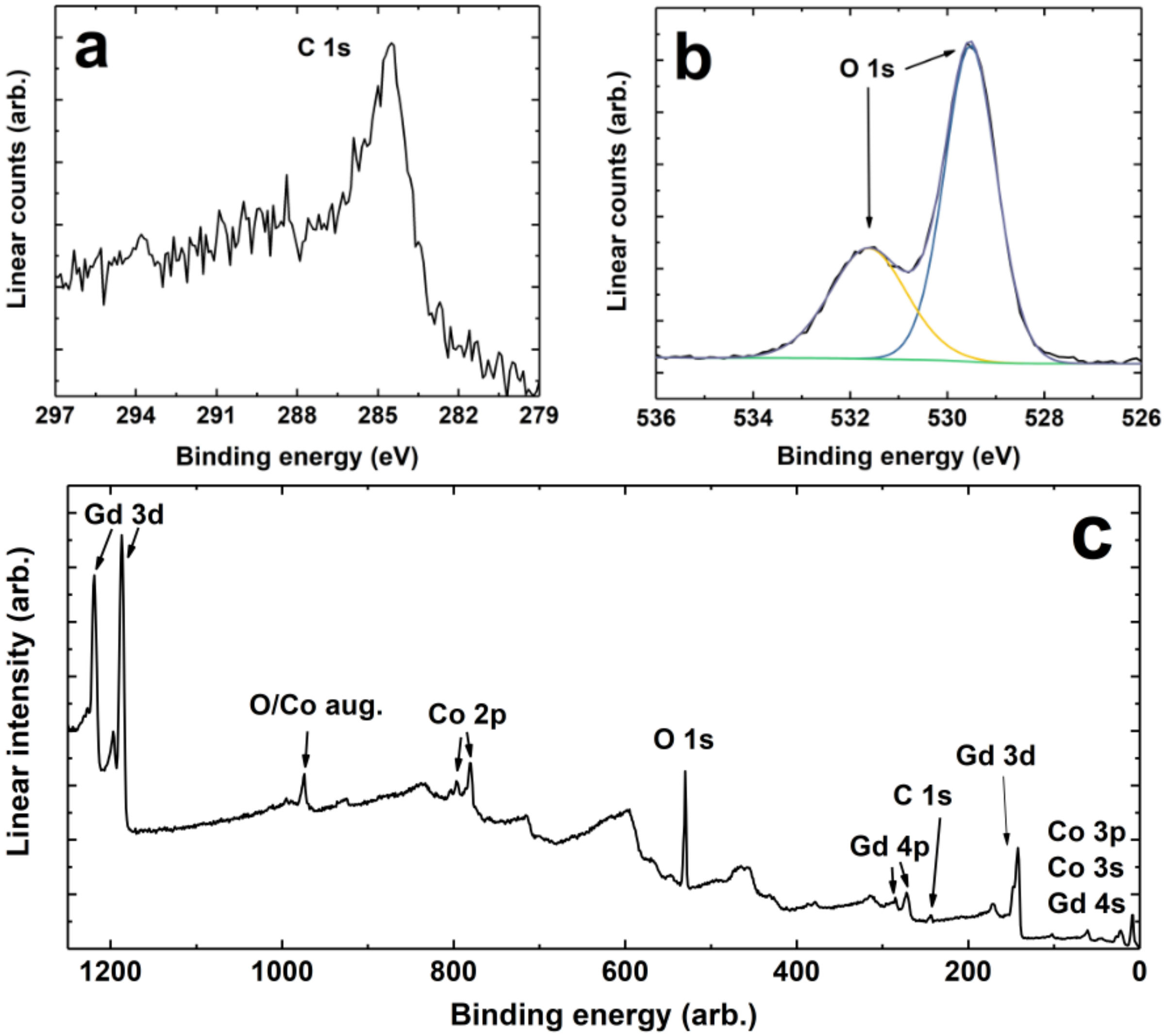


**Figure S3.** (**a**) XPS of C 1s, showing very weak signal pointing towards a very low carbon content. The carbon peak seems to stem from one species at 284.8 eV, attributed to adventitious carbon. (**b**) XPS of O 1s, showing a split peak which is attributed to the two distinct oxygen species in the $GdCoO_3$ structure. It is possible that some O is bonded to C on the surface as carbonate. (**c**) Survey spectra showing identification of Gd, Co, O and carbon species.

© 2019 by the authors. Submitted for possible open access publication under the terms and conditions of the Creative Commons Attribution (CC BY) license (http://creativecommons.org/licenses/by/4.0/).
